# Inflammatory Cytokine Profile Associated with Metabolic Syndrome in Adult Patients with Type 1 Diabetes

**DOI:** 10.1155/2015/972073

**Published:** 2015-07-27

**Authors:** Aldo Ferreira-Hermosillo, Mario Molina-Ayala, Claudia Ramírez-Rentería, Guadalupe Vargas, Baldomero Gonzalez, Armando Isibasi, Irma Archundia-Riveros, Victoria Mendoza

**Affiliations:** ^1^Endocrinology Department, Hospital de Especialidades Centro Médico Nacional Siglo XXI, Instituto Mexicano del Seguro Social (IMSS), Cuauhtémoc 330, Colonia Doctores, 06720 Mexico City, MEX, Mexico; ^2^Experimental Endocrinology Investigation Unit, Hospital de Especialidades Centro Médico Nacional Siglo XXI, Instituto Mexicano del Seguro Social (IMSS), Cuauhtémoc 330, Colonia Doctores, 06720 Mexico City, MEX, Mexico; ^3^Immunochemistry Medical Investigation Unit, Hospital de Especialidades Centro Médico Nacional Siglo XXI, Instituto Mexicano del Seguro Social (IMSS), Cuauhtémoc 330, Colonia Doctores, 06720 Mexico City, MEX, Mexico

## Abstract

*Objective*. To compare the serum concentration of IL-6, IL-10, TNF, IL-8, resistin, and adiponectin in type 1 diabetic patients with and without metabolic syndrome and to determine the cut-off point of the estimated glucose disposal rate that accurately differentiated these groups. *Design*. We conducted a cross-sectional evaluation of all patients in our type 1 diabetes clinic from January 2012 to January 2013. Patients were considered to have metabolic syndrome when they fulfilled the joint statement criteria and were evaluated for clinical, biochemical, and immunological features. *Methods*. We determined serum IL-6, IL-8, IL-10, and TNF with flow cytometry and adiponectin and resistin concentrations with enzyme linked immunosorbent assay in patients with and without metabolic syndrome. We also compared estimated glucose disposal rate between groups. *Results*. We tested 140 patients. Forty-four percent fulfilled the metabolic syndrome criteria (*n* = 61), 54% had central obesity, 30% had hypertriglyceridemia, 29% had hypoalphalipoproteinemia, and 19% had hypertension. We observed that resistin concentrations were higher in patients with MS. *Conclusion*. We found a high prevalence of MS in Mexican patients with T1D. The increased level of resistin may be related to the increased fat mass and could be involved in the development of insulin resistance.

## 1. Introduction

Type 1 diabetes mellitus (T1D) is an autoimmune disease characterized by beta cell destruction [1]. Its incidence has increased by ~3% per year in children under the age of 5 and represents 10% of all the cases of diabetes [2]. In Mexico, the incidence of T1D among children under 19 years of age is 6.2 per 100,000 insured cases in the Instituto Mexicano del Seguro Social (IMSS), the largest social health care provider throughout the country [3]. Our country has a very high prevalence of obesity worldwide in all age groups, and it also affects patients with T1D [4]. Metabolic comorbidities associated with increased weight, such as hypertension or dyslipidemia, are also very common among the Mexican population [5]. Some cohorts have previously described T1D patients with features of insulin resistance and metabolic syndrome [6]. This association, sometimes called “double diabetes” [7], is being actively studied on the basis that each entity may increase cardiovascular morbidity on its own and theoretically generate a synergic and detrimental effect on mortality [8] due to cardiovascular diseases, despite intensive glycemic control [9].

Depending on the population studied and the criteria used, metabolic syndrome (MS) has been reported in 8 to 43% of the T1D patients [6, 10, 11]. The MS components, usually associated with an increased cardiovascular risk, were highly prevalent in patients with T1D and vascular comorbidities [8]. An additional source of controversy is the difficulty to define metabolic syndrome in patients with T1D; since all patients are diabetic, this may represent a selection bias and possibly overdiagnosis; also, the most important feature of MS (insulin resistance) is not part of the pathogenesis of T1D and, finally, the use of exogenous insulin in T1D makes it impossible to predict insulin resistance with simple methods such as the HOMA-IR index calculation. In order to solve these problems, different authors have proposed methods to assess insulin sensitivity such as eGDR (estimated glucose disposal rate), but its utility in patients with T1D and MS has not been determined and since it is based on the measurements of waist-to-hip ratio, it has been suggested that cut-off points for each population need to be validated. In groups with predominantly Hispanic and overweight populations, assessing these cut-offs is even more necessary.

The pathophysiology of the increased cardiovascular risk associated with MS in T1D is complex and not yet completely understood. A wide range of cardiac diseases have been associated with inflammation and cytokine modulation. Type 1 diabetic patients consistently report higher levels of fibrinogen, interleukin-6 (IL-6), C-reactive protein, and tumoral necrosis factor-alpha (TNF-*α*) compared to nondiabetic patients [12, 13]; however these studies were not designed to prove differences between patients with and without MS. On the other hand, additional studies have demonstrated that patients with type 2 diabetes have similar inflammatory profiles, but the general assumption was that their elevated cytokines were generated by insulin resistance and increased adiposity, since some of them decreased significantly once the patients lost weight [14]. Resistin is a proinflammatory cytokine previously considered to be responsible for the obesity-mediated insulin resistance [15, 16]. This cytokine has been found to be elevated in lean T1D patients, but the clinical significance of this elevation has yet to be elucidated. Given these controversial reports and the lack of information regarding patients with double diabetes, especially in high risk populations such as ours, we aimed to compare the inflammatory profile of patients with T1D with and without MS.

## 2. Patients and Methods

We performed a cross-sectional evaluation of all patients in the type 1 diabetes clinic from January 2012 to January 2013 (Hospital de Especialidades Centro Médico Nacional Siglo XXI, a tertiary care referral center). We included patients that were 18 years of age or older at the time of the study and had at least 3 visits per year to the clinic, no infections recorded in the 3 months prior to the study, a normal complete blood count and urinary analysis, and no change in insulin dose in the last 3 months. Patients with incomplete records, poor treatment adherence, clinical or biochemical data of infection, end-stage renal disease, evidence of autoimmune diseases (except for treated primary hypothyroidism), and primary dyslipidemias were excluded. The study completed all the requirements by local ethics committee and was conducted in accordance with the Declaration of Helsinki. The protocol's nature and the aim of the study were fully explained to the subjects, who gave their written consent.

### 2.1. Diagnostic Criteria for MS

Patients were considered to have MS when they presented 3 or more of the joint statement criteria from the American Heart Association/National Heart Lung and Blood Institute (AHA/NHLBI) and the International Diabetes Federation (IDF) [17]: serum triglycerides >150 mg/dL (1.7 mmol/l) or patients receiving treatment for hypertriglyceridemia, serum high-density lipoprotein cholesterol (c-HDL) <40 mg/dL (1.03 mmol/l) in men or <50 mg/dL (1.29 mmol/l) in women or a previously treated dyslipidemia, arterial blood pressure >130/85 mmHg in two different determinations or if the patients were receiving treatment with antihypertensive drugs, and waist circumference (WC) >90 cm in men and >80 in women. Since all the patients were under treatment for type 1 diabetes, they all had fasting plasma glucose >100 mg/dL (5.6 mmol/l) at least once.

### 2.2. Anthropometric Measurements

At initial evaluation we registered weight (kg) and height (meters), as well as WC (cm). Using these parameters we evaluated waist-to-height ratio (WHtR) and waist-to-hip ratio (WHR). A single investigator, using the same calibrated instruments, performed all the anthropometric measurements. WC was determined at the middle point between the inferior rim of the last costal arch and the superior rim of the anterosuperior iliac spine. Body mass index (BMI) was calculated with the formula that divides weight by height to the square. We used BMI determination to define weight groups, according to the World Health Organization (WHO) [18] classification. Blood pressure was determined in the left arm, after 10 minutes in a resting position, during a fasting state, without coffee or tobacco ingestion in the last week. The sphygmomanometer was calibrated and values were averaged after 2 different measurements with a 5-minute difference between them.

### 2.3. Biochemical Determinations

Laboratory results were obtained with a 6 mL sample in BD Vacutainer (BD, Franklin Lakes, NJ, USA) and centrifuged at 3150 ×g for 15 minutes, and serum was divided into two aliquots. We analyzed glucose, cholesterol, c-HDL, and triglycerides with a commercially available kit (COBAS 2010 Roche Diagnostics, Indianapolis, USA) using photocolorimetry with spectrophotometer Roche Modular P800 (2010 Roche Diagnostics, Indianapolis, USA). c-HDL samples were treated with enzymes modified with polyethylene glycol and dextran sulphate, analyzed with the same photocolorimetric technique. Glycated hemoglobin (HbA1c) was evaluated by turbidimetric immunoanalysis (COBAS 2010 Roche Diagnostics, Indianapolis, USA). Low-density lipoprotein cholesterol (c-LDL) was calculated with Friedewald formula c-LDL (mg/dL) = CT mg/dL − (c-HDL mg/dL + triglycerides mg/dL/5) if triglycerides were <400 mg/dL [19].

### 2.4. Cytokine Determination

Adiponectin and resistin were analyzed with a commercial kit (Human Adiponectin and Resistin Platinum ELISA tests) using enzyme linked immunosorbent assay (ELISA). Interassay coefficients of variation (%CV) for adiponectin and resistin ranged from 5.8 to 6.9% and from 7.8 to 9.2%, respectively. Interleukin-8 (IL-8), interleukin-6 (IL-6), interleukin-10 (IL-10), and total tumor necrosis factor (TNF) were analyzed with BD Cytometric Bead Array (CBA) Human Inflammation Kit using flow cytometry (BD FACSAria, BD Biosciences, USA). The %CV of IL-8 was 4–7%, %CV of IL-6 was 8–10%, %CV of IL-10 was 8–11%, and %CV of TNF was 8–15%. Detection limits for each assay were 1.5 pg/mL for IL-6, 2.7 pg/mL for TNF, 2.3 pg/mL for IL-10, 2.6 pg/mL for IL-8, 0.24 ng/mL for adiponectin, and 0.26 ng/mL for resistin.

### 2.5. Insulin Resistance Quantification

Insulin resistance was calculated using the estimated glucose disposal rate (eGDR) according to the following formula: 24.31 − (12.22 × waist-to-hip ratio [WHR]) − (3.29 × hypertension [defined as 0 = no, 1 = yes]) − (0.57 × HbA1c). Using this formula, a lower eGDR level indicates greater insulin resistance [20].

### 2.6. Statistical Analysis

Data was analyzed with STATA v.11. Kolmogorov-Smirnov test was used to determine normality. Results are expressed accordingly with means and standard deviations (SD) or medians and interquartile ranges (IQR). To establish associations between quantitative variables, Student's *t*-test or Mann-Whitney *U* test was used. Qualitative variables were associated with *χ* ^2^ or Fisher's test. Additionally, correlations were performed using a Spearman test. Receiver operating characteristic (ROC) curves were used to identify the best cut-off point of eGDR and resistin with area under curve (AUC) and 95% confidence intervals. To evaluate the factors associated with the presence of the MS, a multiple logistic regression model was performed. A *p* < 0.05 was considered to be significant.

## 3. Results

We tested 140 patients during the study period with a median age of 28 years (22–37 years); 70% of them were female. Median time from diagnosis was 17 years (11–25 years). In the whole group, 54% had a WC larger than the recommended for their sex (central obesity), 30% had hypertriglyceridemia, 29% had hypoalphalipoproteinemia, and 19% were hypertensive. According to the WHO classification [18] only 58% of the patients were considered to be in the normal weight range, 33% were overweight (*n* = 46), and 8% were obese (*n* = 11). Using joint statement criteria, 44% of the patients were considered to have MS.  Table 1 compares the baseline characteristics of the groups with and without MS.

Patients with MS are significantly older and have longer evolution of the disease. As it was expected, they also have higher BMI, WC, WHtR, and WHR; their total cholesterol, triglycerides, and c-LDL concentrations were higher while c-HDL was lower; however insulin dose per body weight, glomerular filtration rate, and HbA1c concentration were not different between groups.

### 3.1. Insulin Resistance Quantification

**Figure 1:**
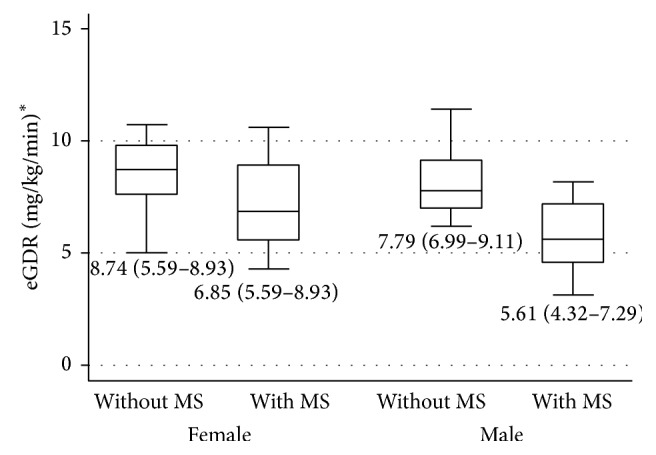
Estimated glucose disposal rate (eGDR) levels in male and female patients with and without MS. ^*^Data is presented as median (IR).

Although there were no significant differences in the insulin requirements between the groups, eGDR levels were lower in patients with MS (6.63 mg/kg/min, IQR 4.79–8.59), when compared to the group without MS (8.42 mg/kg/min, IQR 7.49–9.67) (*p* < 0.001). The lowest eGDR was registered in the male patients with MS; however it was not statistically different from their female counterparts (Figure 1).

We performed a receiver operating characteristic (ROC) curve and Youden index for assessing optimal eGDR cut-off point for detecting MS in T1D patients. An eGDR level below 7.32 mg/kg/min showed 80% sensitivity and 66% specificity for MS diagnosis, with an area under curve (AUC) of 0.743 (IC 95% 0.648–0.839) (*p* < 0.001).

### 3.2. Inflammatory Profile

Table 2 shows the inflammatory profile assessed in all T1D patients. We observed that IL-8 and resistin concentrations were higher in patients with MS; however IL-8 levels were not statistically different between groups. We did not find any difference in the cytokine levels when comparing groups according to sex, HbA1c groups (<7%, 7-8%, or >8%), or obesity grades (BMI rate normal, overweight, or obesity) (data not shown). Resistin was the only cytokine that showed a significant difference between the groups with and without MS. A ROC curve identified that a cut-off point of 1108 pg/mL detects MS with a sensitivity of 71% and specificity of 56%, with an AUC of 0.68 (0.55–0.81). We also quantified correlation coefficients between these cytokines and observed a positive correlation between IL-8, IL-6, IL-10, and resistin in patients with and without MS (Table 2). As it was expected, the resistin concentrations also correlated with IL-6 (*r* = 0.398, *p* = 0.001) and with TNF (*r* = 0.533, *p* < 0.001). Only the group with MS showed a positive correlation in these cytokines: resistin versus IL-6 (*r* = 0.532, *p* = 0.001) and versus TNF (*r* = 0.582, *p* = 0.001). When we correlated the different cytokines, we found a positive correlation between IL-10 and IL-6 (*r* = 0.676, *p* ≤ 0.001) and between IL-10 and TNF (*r* = 492, *p* = 0.001); other cytokines did not show any significant correlation.

Many patients' results were found in the lowest detection limit for each cytokine. When we consider these patients as “undetectable” and compare them with the patients that reported higher cytokines (detectable), there are significant differences between the percentages of patients in these categories when dividing according to resistin levels. In the group with high resistin level (>1108 pg/mL) we found that IL-6 was detectable (>1.5 pg/mL) in 39.4% versus only 11.5% of detectable IL-6 in the group of patients with low resistin (*p* = 0.03). Furthermore TNF was detectable (levels higher than 2.7 pg/mL) in 47% of patients with high resistin versus 4% in patients with low resistin (*p* < 0.001).

### 3.3. Risk Factors for MS Development

We assessed factors associated with the presence of MS using a multiple logistic regression analysis. Only familiar history of obesity showed an OR 2.06 (CI 95% 1.02–4.14). Neither familial history of diabetes, dyslipidemia, or hypertension nor adherence to diet, regular exercise, or smoking was related to the presence of MS.

## 4. Discussion

The presence of features associated with MS in patients with T1D has been associated with an increased risk for macro- and microvascular complications [21]. However, the etiopathogeny and the long-term clinical significance of this association need yet to be established. The prevalence of MS in patients with T1D varies widely depending on the studied population and criteria used for diagnosis [22]. Despite the controversy generated by the difficulties of diagnosing MS in patients with T1D, the joint statement criteria seem to be the most adequate and widely used in these patients. The inclusion of a WC cut-off helps detecting MS in different ethnic and body fat distribution groups, such as ours where traditional anthropometric and biochemical data is not enough to correctly define high risk patterns. In our study, using these criteria, we found that more than 40% of our T1D patients show additional metabolic disturbances that are associated with higher cardiovascular risk. It is known that age plays an important role in the development of MS and our patients with MS were significantly older than the ones without MS; however, this difference was not associated with any significant changes in the biochemical or inflammatory profile.

We report the highest frequency of MS in a group of patients with T1D to our knowledge. Although it reflects the worrying epidemiologic tendencies of the general population in our country, we should also consider that, in addition to the MS diagnosis, the presence of a long-standing autoimmune/inflammatory disorder in adult patients with poorly controlled diabetes could mean a significant increase in cardiovascular mortality and microvascular complications in the near future. Interestingly, the tendency to report higher frequencies of obesity or other components of the MS in patients with T1D is not exclusive of the Mexican population, since recent reports mention an increased frequency of central obesity and dyslipidemia in different cohorts, despite their attempts to strictly control diabetes [23, 24].

Additional studies are required in order to find markers and cut-off points that may unequivocally classify T1D patients with MS and high cardiovascular risk. Cytokines are some of the biomarkers that have been actively studied in the last few years, since some patterns of increased and decreased cytokine levels correlate with specific inflammatory states and morbidities. Obesity, cardiovascular disease, and even type 2 diabetes seem to generate different cytokine profiles that could help to explain the physiopathology of these diseases and even serve as prognostic factors or controls for therapeutic interventions in the future [25–27]. Cytokine levels have not been previously evaluated in adult patients with T1D with an intention to establish differences between patients with and without MS. Our results show that most of the traditional cytokines measured in other studies are similar in both groups, for proinflammatory and anti-inflammatory interleukins. This may reflect a similar state of inflammation in both groups. Additionally, we found a positive correlation between IL-10 (an anti-inflammatory cytokine) with TNF and IL-6 (usually proinflammatory cytokines). This correlation has been found in certain pathologies such as thyroid cancer [28], stroke [29], lupus, and Sjögren syndrome [30], where the inflammatory profile may be altered from their basal state. Adiponectin showed no significant correlations with any other cytokine. Based on the apparently paradoxical correlations between pro- and anti-inflammatory cytokines, we support previous authors' ideas that IL-10 may have differential actions in different stages of the inflammatory response; however only longitudinal studies may be able to solve these questions in vivo. In our study, neither age nor time from diagnosis of T1D translated into differences in cytokine levels.

We consider that resistin may be an important inflammatory cytokine in these patients. It is synthesized mainly by adipose tissue and seems to generate insulin resistance through an inhibitory effect on CD36, fatty acid transport protein 1 (FATP1), acetyl-coA carboxylase, and AMP-activated protein kinase *α*. Furthermore, resistin has been shown to regulate gene expression of TNF-*α* and IL-6 via nuclear factor-k*β* (NF-k*β*) [31]. These characteristics make it a unique marker that may be strongly related to the effect of excess weight and weight changes on the inflammatory profile. Our study shows that resistin is positively correlated to other cytokines in the whole patient group, reflecting the inflammatory state generated by T1D; however, when we divided the group, only the patients with MS consistently showed this positive correlation, while the patients without MS showed a small and nonsignificant correlation. Previous studies by Fehmann and Heyn reported no significant differences in the resistin levels between patients with or without MS; however we should note that their population was not so markedly obese [32].

Our data also differs from the findings of Timar et al. [33]. They found that all proinflammatory cytokines were high and the anti-inflammatory cytokines were low. However, the basal characteristics of their groups also seem radically different and their patients with MS have higher HbA1c and insulin dose/kg than the group without MS. Considering the possible differential effect of cytokines according to age and disease severity, these differences may account for the results in the cytokine levels. Also the methods used to measure cytokines are different and resistin was not considered in their analysis.

We suggest that adding resistin to the cytokine panel in the study of MS will continue proving to be relevant in future reports, especially in populations like ours, where a large proportion of the patients show an increased fat mass.

Traditionally, the definition of insulin resistance in T1D has been proven difficult. Insulin clamps are the gold standard for these studies; however these procedures are invasive and not useful in the everyday clinical setting. The use of eGDR has been recently advocated as a useful way to assess insulin resistance in this group [34]. Its advantages as a validated tool are limited by the fact that race and ethnicity play a significant role in insulin sensitivity. Previous papers published by different authors show that lower eGDR scores correlate with larger insulin resistance, but no specific cut-off point has been established so far. We observed that a cut-off point of 7.32 mg/kg^−1^/min^−1^ predicts MS development with a sensitivity of 80% and specificity of 66%, in a group consisting entirely of Mexican mestizos. Despite the fact that this cut-off may not be adequate for other ethnicities, our results show clearly that the patients with MS have the lowest eGDR scores and therefore, they present with more insulin resistance, despite the apparently similar requirements of insulin. Cytokines, and specially resistin, may be some of the factors modulating insulin resistance in T1D and therefore warrant additional studies.

Our study has some limitations. The cross-sectional study design prevents us from identifying the effect of time on MS. Also, we consider that the low (often undetectable) cytokine levels observed could be related to a dilution effect or a low detection power in our methods. We believe that future studies determining cytokines mRNAs from mononuclear cells or adipose tissue may prove more useful; however, the study of mRNA may also be limited, since one must consider the possible effect of posttranscriptional regulation of RNA-binding proteins (i.e., tristetraprolin, adenine- and uridine-rich element (ARE)/poly(U) binding degradation factor 1, ZCCHC11 and regnase-1) or certain microRNAs [35]. Additionally, although we assessed the main pro- and anti-inflammatory cytokines with detectable differences only in resistin, other pathways may be important in the regulation of cytokines, for example, IL-17, pathways related to transforming growth factor-beta (TGF-*β*) and endothelial activity through E-selectin, vascular cell adhesion molecule (VCAM-1), or von Willebrand factor (vWF).

Prospective, long-term studies with highly sensitive determinations of multiple cytokines may help understand the patterns of inflammation present in patients with double diabetes and may also help us differentiate them from other inflammatory states and stages of the various diseases related to cardiovascular morbidity. Identifying the patients with the highest risk of cardiovascular disease and the mechanisms underlying their etiopathogeny may be some of the final goals of these lines of investigation.

## 5. Conclusions

We found a high prevalence of MS in Mexican patients with T1D. This condition could be related to the high prevalence of obesity and dyslipidemia already seen in our population, but it may represent an additional risk factor for cardiovascular disease in T1D patients. The increased level of resistin level and its correlation to other inflammatory cytokines may be related to the increased fat mass and could be involved in the development of insulin resistance observed in our patients. Although we did not observe any differences in other cytokines, we consider that more studies are required to fully understand the inflammatory profile in patients with double diabetes. Finally, we determine an optimal cut-off point for diagnosis of MS in our group with a high sensitivity and middle specificity. To our knowledge this seems to be the first study that determines insulin resistance through eGDR in Mexican population.

## Figures and Tables

**Table 1:** Baseline characteristics of the population comparing patients with and without MS.

Parameter	With MS (*n* = 61)	Without MS (*n* = 79)	*p*
Age, years (median, IR)	34 (24–42)	26 (20–32)	0.001^*^

Evolution, years (median, IR)	19 (12.5–27.5)	16 (10–22)	0.021^*^

WC, cm (mean ± SD)	90.3 ± 10	80 ± 9.2	<0.001^†^
Male	92.3 ± 11.31	85.9 ± 7.61	0.05^†^
Female	89.5 ± 9.56	77.2 ± 8.65	<0.001^†^

BMI, kg/m^2^ (mean ± SD)	26.1 ± 3.83	23.5 ± 3.09	<0.001^†^
Male	25.7 ± 4.1	23.8 ± 3.3	0.124^†^
Female	26.3 ± 3.7	23.3 ± 3	<0.001^†^

WHR (mean ± SD)	0.90 ± 0.06	0.87 ± 0.07	0.001^†^
Male	0.95 ± 0.04	0.90 ± 0.06	0.016^†^
Female	0.90 ± 0.07	0.86 ± 0.06	0.009^†^

WHtR (median, IR)	0.54 (0.5–0.6)	0.48 (0.45–0.52)	<0.001^*^

Systolic blood pressure, mmHg (median, IR)	110 (100–120)	110 (100–120)	NS

Diastolic blood pressure, mmHg (mean ± SD)	68.7 ± 8.43	67 ± 8.34	NS

Insulin dose, units (median, IR)	54 (43–72)	52 (42–60)	NS
U/body weight (kg) (median, IR)	0.67 (0.54–0.93)	0.83 (0.63–1.04)	

Glomerular filtration rate, mL/min (median, IR)	77.9 (30.5–97)	76 (46.9–98)	NS

Fasting glucose, mg/dL (median, IR)	150 (88–220)	140 (94–238)	NS

HbA1c, % (median, IR)	9 (8–10)	8 (8–10)	NS

Cholesterol, mg/dL (median, IR)	198 (163–240)	175 (153–206)	0.005^*^

Triglycerides, mg/dL (median, IR)	164 (113–241)	89 (65–119)	<0.001^*^

c-HDL, mg/dL (median, IR)	46 (37–59)	57 (47–67)	<0.001^*^
Male	44 (35.7–52.7)	46.5 (42.2–54)	0.23
Female	51 (37–63)	61 (53–71)	<0.001^*^

c-LDL, mg/dL (median, IR)	112 (94.5–137.5)	103 (80–116)	0.018^*^

IR: interquartile range, WC: waist circumference, BMI: body mass index, WHR: waist-to-hip ratio, WHtR: waist-to-height ratio, HbA1c: glycated hemoglobin, c-HDL: cholesterol associated with high-density lipoprotein, c-LDL: cholesterol associated with low-density lipoprotein.

^*^Mann-Whitney *U* test; ^†^Student's *t*-test.

**Table 2:** Inflammatory profile in total T1D population and in patients with and without MS.

Parameter	T1D population (*n* = 140)	Patients with MS (*n* = 61)	Patients without MS (*n* = 79)	*p* ^*^
IL-8, pg/mL (median, IR)	18.2 (8.68–41.22)	24.6 (12.65–46.5)	13.4 (7.0–34.4)	0.064
IL-6, pg/mL (median, IR)	1.5 (1.5–3.2)	1.5 (1.5–4.7)	1.5	NS
IL-10, pg/mL (median, IR)	2.3 (2.3–3)	2.3 (2.3–4.4)	2.3	NS
TNF, pg/mL (median, IR)	2.7 (2.7–4.8)	2.7 (2.7–7.8)	2.7	NS
Adiponectin, pg/mL (median, IR)	9.4 (5.7–15.5)	8.9 (5.4–14.9)	9.4 (6.6–15.6)	NS
Resistin, pg/mL (median, IR)	1180.3 (775.8–1896.8)	1627.4 (838.8–2233.8)	1055 (631.8–1459.2)	0.010
%Patients with resistin >1108 pg/mL	55%	71%	44%	0.021

IR: interquartile range, T1D: type 1 diabetes, MS: metabolic syndrome, NS: not significant, IL-8: interleukin-8, IL-6: interleukin-6, IL-10: interleukin-10, and TNF: tumor necrosis factor. ^*^Comparing patients with and without MS, using Mann-Whitney *U* test.
